# Symptom Clusters and Quality of Life in Cervical Cancer Patients Receiving Concurrent Chemoradiotherapy: The Mediating Role of Illness Perceptions

**DOI:** 10.3389/fpsyt.2021.807974

**Published:** 2022-01-31

**Authors:** Lan Zhang, Jia Wang, Tangzhen Chen, Min Tian, Qimin Zhou, Jianhua Ren

**Affiliations:** ^1^Department of Nursing, West China Second University Hospital, Sichuan University/West China School of Nursing, Sichuan University, Chengdu, China; ^2^Key Laboratory of Birth Defects and Related Diseases of Women and Children (Sichuan University), Ministry of Education, Chengdu, China; ^3^Department of Health Psychology, School of Nursing, Shandong University, Jinan, China

**Keywords:** cervical cancer, symptom clusters, illness perceptions, quality of life, mediating role

## Abstract

**Objectives:**

Although studies shows that symptom clusters and illness perceptions are negatively associated with quality of life (QoL), it is unclear how these variables of cervical cancer patients who receive concurrent chemoradiotherapy (CCRT) relate to each other. This study aimed to identify the symptom clusters in cervical cancer patients who receive CCRT and evaluate the mediating effect of illness perceptions on the relationship between symptom clusters and QoL.

**Methods:**

A cross-sectional survey was conducted on 286 cervical cancer patients receiving CCRT from October 2019 to October 2020. M.D. Anderson Symptom Inventory, Brief Illness Perception Questionnaire, and Functional Assessment Cancer Therapy-Cervix were applied to investigate the symptom clusters, illness perceptions and QoL of the participants, respectively. Exploratory factor analysis was conducted to identify symptom clusters. The relationships among symptom clusters, illness perceptions, and QoL were analyzed with the structural equation modeling.

**Results:**

A total of four symptom clusters were identified, including psychological status symptom cluster, therapy side-effect symptom cluster, sickness symptom cluster, and gastrointestinal symptom cluster (χ^2^ = 1,552.282, Df = 78, *P* < 0.001). Symptom clusters, illness perceptions, and QoL were significantly correlated. Symptom clusters had significant direct (β = −0.38, *P* < 0.001) and indirect effects (β = −0.21, *P* < 0.001) on QoL.

**Conclusion:**

Illness perceptions played a significant mediating role between symptom clusters and QoL in cervical cancer patients receiving CCRT. Strategies like prompting effective symptom management for the purposes of alleviating illness perceptions may contribute to improving their QoL.

## Introduction

According to the Global Cancer Statistics Report, there were an estimated 570,000 new cases and 311,000 deaths by cervical cancer in 2018 worldwide (1). With the deepening understanding of the etiologic link between carcinogenic human papillomavirus (HPV) and cervical cancer, new approaches of primary (HPV vaccination) and secondary prevention (screening for HPV and treating precancerous lesions) have emerged (2). Advancements in early detection and treatment for cervical cancer have improved the overall survival rates of patients and some of them had even achieved the permanent survival phase (3, 4). Survival is no longer the single purpose of treatment for cervical cancer, and quality of life (QoL) has become a substantial concern for long-term post-treatment life, which in turn would impact the outcomes of illness. It is imperative to take measures to maximize the QoL of cervical cancer patients.

National Comprehensive Cancer Network guidelines reported that 60% to 80% of patients with locally advanced cervical cancer can achieve satisfied medical outcomes through concurrent chemoradiotherapy (CCRT) (5). However, the long-term side effects and late toxic reactions of this treatment are common, which would immensely compromise the patients' QoL. Previous studies indicated that patients, who were receiving chemotherapy, suffered from approximate 10 kinds of painful symptoms. The cervical cancer women who were undertaking CCRT would suffer more complications and side effects than those receiving surgery alone (6–9). The multiple co-occurring symptoms has been proved to be one of the most important factors that would influence the QoL among cancer survivors negatively (10). However, most studies on cervical cancer placed their focus on the impact of certain single symptom rather than symptom clusters on QoL (11–13).

Symptom clusters are defined as three or more interrelated concurrent symptoms (14). Those symptoms may share similar mechanisms or have additive or synergistic effects (14, 15). Increasing evidence has demonstrated a greater effect of symptom clusters than the mere sum of single ones on the QoL in cancer survivors (16–18). It is essential to learn about the impact of symptom clusters on the QoL of various cancer survivors, including cervical cancer ones, which may become the reference for intervention designing for cervical cancer patients. Unfortunately, little is known about the symptom clusters in cervical cancer patients undergoing CCRT. Wang et al. extracted four symptom clusters among Chinese cervical cancer patients and identified that therapeutic regimen might have significant effect on symptom clusters (19). Identifying the type of symptom clusters and the association between symptom clusters and the QoL is an essential for promoting health outcomes.

Considering that the QoL is the comprehensive assessment of patients' physical and mental health, it is necessary to consult validated theoretical frameworks such as the Common-Sense Model (CSM) (20, 21). According to Leventhal et al., the CSM proposes that when confronted with an illness or a threat to health, individuals create cognitive and emotional representations, including identity, timeline, consequences, causes, and controllability. As the core element of this model, illness perceptions were defined as patients' views, beliefs, and emotional responses concerning illness and treatment (22). Studies have linked illness perceptions to psychological distress and poor QoL (23–26). The increased threatening illness perceptions experienced by gynecological cancer survivors would result in poorer physical and psychosocial outcomes (23). Sayilan et al. also confirmed that for patients with a diagnosis of cancer, QoL, physical and social well-being improved as illness perceptions decreased (24). Pereira et al. reported that the associations of breast symptoms between psychological disturbance and QoL are completely mediated by illness perceptions (27). Similar results have been found in other chronic diseases, where the severity of symptoms affects the QoL not only directly, but also indirectly through illness perceptions (28, 29). Notably, although there has been increased interest in the illness perceptions of cancer survivors, research regarding this variable in cervical cancer patients is limited (30). Thus, when multiple symptoms are co-occurring, the relationships among symptom clusters, illness perceptions, and QoL in cervical cancer patients remain unclear.

Based on the CSM and previous findings, our study focus on the antecedents and consequences of illness perceptions in cervical cancer patients undergoing CCRT. The purposes of this study are (1) to identify symptom clusters and (2) to evaluate the mediating effect of illness perceptions on the relationship between symptom clusters and QoL.

## Methods

### Participants

Patients with cervical cancer undergoing CCRT were recruited by a non-probability convenient sampling procedure from the medical oncology inpatient units of three hospitals in China. The inclusion criteria were as follows: (a) diagnosis of cervical cancer by histopathological evaluation; (b) being at least 18 years old; (c) having already received CCRT; (d) able to voluntarily participate in the investigation and sign informed consent. Participants who were unable to complete the survey due to severe physical diseases, psychological distress, or cognitive impairment were excluded.

Generally, the minimum sample size of 200 participants is sufficient for structural equation modeling (SEM) or 5 to 20 times the number of variables to be estimated (31). Thus, a total of 286 participants met the aforementioned rules.

### Study Design and Procedure

A cross-sectional study was conducted between October 2019 and October 2020. To collect study data, three trained interviewers conducted face-to-face interviews using unified instructions and procedures. Before recruitment and investigation, the purpose and content of this study were explained in detail to the respondents. The eligible candidates who met all the inclusion criteria and who voluntarily participated in this study signed a written informed consent form. Each volunteer typically took approximately 15 to 20 min to complete the questionnaire. A total of 300 patients were invited to participate in our research, of whom 295 (98.3%) completed the questionnaires. Nine unqualified questionnaires were excluded from the analysis because of more than 10% of unanswered items. Finally, vailed questionnaires were collected from 286 individuals, with an effective rate of 95.3%. Ethical approval was received from the Ethics Committee of the author's university (2020-R-059). This study was carried out in compliance with the STROBE statement and the Helsinki Declaration.

### Measures

#### Sociodemographic and Clinical Characteristics

According to the literature reviews, the self-designed questionnaire was developed to evaluate sociodemographic information (age, Body Mass Index, education, occupation, marital status, reproductive history, monthly income per capita, and residence) and clinical characteristics (course of disease, tumor stage, and tumor type).

#### M.D. Anderson Symptom Inventory (MDASI)

The original section of this scale consists of 19 items, which are divided into two parts. The first part (13 items) and the second part (6 items) evaluate the severity of symptoms and interference of these symptoms during daily activities in the past 1 to 2 days, respectively. Each item ranges from 0 to 10 (with 0 indicating “no symptom or interference” and 10 indicating “extreme symptom or interference.” The two parts are interpreted separately and the average score for each item is calculated. Individual item severity and interference were rated using the following method: none (0), mild (1–4), moderate (5, 6), and severe (≥7) (32). The Chinese version of MDASI, translated and revised by Wang et al., has shown good reliability and validity in Chinese cancer patients (33). In this study, the internal consistency coefficient (Cronbach's α) for the scale was 0.843.

#### Brief Illness Perception Questionnaire (BIPQ)

The 9-item BIPQ was adopted to assess patients' cognitive performance and emotional representations of disease (34). The instrument consists of five items to evaluate cognitive illness representations (perceived consequences, timeline, personal control, treatment control, identity), two items to evaluate emotional representations (concern and emotional response), one item to evaluate illness comprehensibility, and an open-ended question to explore causes of the disease (not considered in this current research). Items are rated using an 11-point Likert-type scale (range 0–10), with higher scores reflecting more threatening illness perceptions. The Chinese version of BIPQ has been tested and applied to cancer patients (35). In this study, the Cronbach's α coefficient of BIPQ was 0.802.

#### Functional Assessment Cancer Therapy-Cervix (FACT-Cx)

FACT-Cx consists of the Functional Assessment of Cancer Therapy-General (FACT-G, 27 items) plus cervical cancer subscale (CCS, 15 items) to yield a comprehensive assessment of QoL (36). The questionnaire comprises 42 items on a 5-point Likert scale ranging from 0 to 4 and is categorized into the following five dimensions: physical well-being, social/family well-being, emotional well-being, functional well-being, and CCS. A total score is the sum of all 42 items and ranges from 0 to 168, with a higher total score representing a better QoL. The Chinese version of FACT-Cx has been demonstrated to be reliable and valid in cervical cancer patients (37). In our study, the Cronbach's α coefficient of FACT-Cx was 0.928.

### Statistical Analyses

Data analyses were performed by statistic package for social science (IBM SPSS, version 26.0 for Windows, Armonk, NY, USA), and AMOS software (IBM SPSS AMOS, version 22.0, Chicago). The variables contained in the model were normally distributed (tested by inspecting frequency histograms). Results were presented as mean ± standard deviation (SD) for continuous data and as count for categorical data.

Exploratory factor analysis (EFA) was conducted to extract symptom clusters (38). We then performed principal component analysis and varimax rotation with Kaiser normalization to identify the factor structure. Factors with an eigenvalue >1.0 were retained, and loadings of symptoms on factors ≥0.5 were permitted. Moreover, the Kaiser–Meyer–Olkin (KMO) measure and Bartlett's test of sphericity were determined to confirm good sampling adequacy for factor analysis.

Pearson correlation analysis was conducted to explore relations between variables. Regression analysis was performed to select the control variables entering the SEM and assess the influence of sociodemographic and clinical characteristic variables on QoL.

Subsequently, a mediation model was constructed and tested, in which illness perceptions were placed as potential mediators between symptom clusters and QoL. Path analysis was performed within an SEM framework and model parameters were estimated by the maximum likelihood method. We generated a bootstrap approach with 5,000 samples to estimate 95% bias-corrected confidence intervals (CI). The mediation effect can be inferred significant at a *p*-value of 0.05 if the 95% CI does not include 0. Model fit was assessed by modification index and goodness of fit. Indices for the goodness of fit included chi-square to degrees of freedom ratio (χ^2^/df), goodness-of-fit index (GFI), adjusted goodness-of-fit index (AGFI), Tucker-Lewis fit index (TLI), comparative fit index (CFI), root mean square error of approximation (RMSEA). In general, the model provided an acceptable fit if χ^2^/df was ≤ 3, RMSEA was ≤ 0.08, and the rest of indices were ≥0.90 (39). All tests were two-sided and the statistical significance level for all analyses was defined as *P* < 0.05.

## Results

### Sample Characteristics

A total of 286 cervical cancer patients receiving CCRT met eligibility criteria and the sample characteristics are presented in Table 1. The average age of participants was 50.46 years (SD = 10.05), 93.4% of the respondents were married, 53.2% had a monthly per capita income of <3,000 yuan, and 82.9% lived in rural areas or towns. The patients with tumor stage III or IV accounted for 38.1%, and the course of disease (mean months = 8.24, SD = 14.33) <3 months accounted for 51.7%.

**Table 1:** Sociodemographic and clinical characteristics of the patients (*N* = 286).

**Variable**	**Category**	***n* (%)**
Age (years)	<35	25 (8.7)
	35–55	175 (61.2)
	>55	86 (30.1)
Body mass index (kg/m^2^)	<18.5	5 (1.8)
	18.5–23.9	178 (62.2)
	24–27.9	93 (32.5)
	≥28	10 (3.5)
Education	≤ Junior high school	192 (67.1)
	High school	75 (26.2)
	≥College	19 (6.7)
Employed	Yes	214 (74.8)
	No	72 (25.2)
Marital status	Married	267 (93.4)
	Single	10 (3.5)
	Divorced/widowed	9 (3.1)
Had given birth to at least 1 child	Yes	273 (95.5)
	No	13 (4.5)
Monthly income per capita (RMB)	<3,000	152 (53.2)
	3,000–5,000	110 (38.4)
	>5,000	24 (8.4)
Residence	Rural areas	138 (48.3)
	Towns	99 (34.6)
	Cities	49 (17.1)
Course of disease (months)	<3	148 (51.7)
	3–6	68 (23.8)
	>6	70 (24.5)
Tumor stage	I	74 (25.9)
	II	103 (36.0)
	III/IV	109 (38.1)
Tumor type	Squamous cell carcinoma	211 (73.8)
	Adenocarcinoma	63 (22.0)
	Others	12 (4.2)

*SD, Standard Deviation; RMB, Ren Min Bi*.

### Symptom Prevalence, Severity, and Interference

The five most commonly occurring symptoms were fatigue (80.8%), disturbed sleep (79.7%), pain (76.9%), sadness (75.5%), and nausea (73.4%). The five most severe symptoms included fatigue (mean ± SD, 4.05 ± 2.05), nausea (3.60 ± 2.24), disturbed sleep (3.39 ± 1.76), sadness (3.46 ± 2.04), and pain (3.20 ± 1.78). The three most serious items in the symptom interference were “work” (5.17 ± 2.43), “mood” (4.14 ± 2.08), and “general activity” (3.56 ± 2.25). The severity and prevalence of symptoms and interference are set out in Table 2.

**Table 2:** The severity and prevalence of symptoms and interference (*N* = 286).

**MDASI**	**Severity mean ± SD**	**Prevalence *n* (%)**	**Range**
**Symptom**			
Fatigue	4.05 ± 2.05	231 (80.8)	0–8
Dry mouth	2.61 ± 1.45	161 (56.3)	0–7
Nausea	3.60 ± 2.24	210 (73.4)	0–9
Disturbed sleep	3.39 ± 1.76	228 (79.7)	0–8
Vomiting	2.72 ± 1.75	156 (54.5)	0–7
Lack of appetite	2.62 ± 1.55	162 (56.6)	0–7
Pain	3.20 ± 1.78	220 (76.9)	0–8
Sadness	3.46 ± 2.04	216 (75.5)	0–7
Numbness	2.18 ± 1.38	115 (40.2)	0–6
Drowsiness	2.22 ± 1.42	127 (44.4)	0–6
Shortness of breath	2.07 ± 1.40	117 (40.9)	0–6
Distress	3.09 ± 2.03	200 (69.9)	0–8
Forgetfulness	1.97 ± 1.43	116 (40.6)	0–5
**Interference**			
Work	5.17 ± 2.43	235 (82.2)	0–10
Mood	4.14 ± 2.08	232 (81.1)	0–10
General activity	3.56 ± 2.25	211 (73.8)	0–9
Walking	2.31 ± 1.84	137 (47.9)	0–9
Enjoyment of life	1.80 ± 1.87	97 (33.9)	0–8
Relationships with others	1.27 ± 1.64	66 (23.1)	0–9

*MDASI, M.D. Anderson Symptom Inventory; SD, Standard Deviation*.

### Symptom Clusters

KMO and Bartlett tests of sphericity were performed before applying a Factor Analysis. The results showed that these data are suitable tor EFA (KMO = 0.832, χ^2^ = 1,552.282, Df = 78, *P* < 0.001). Principal component analysis was carried out to extract common factors with eigenvalues >1.0. Four symptom clusters were identified, with a cumulative variance contribution rate reaching 70.7%. We named symptom clusters according to the items contained in each symptom cluster. They were named as “psychological status symptom cluster” (distress, sadness, lack of appetite, and fatigue), “therapy side-effect symptom cluster” (forgetfulness, shortness of breath, drowsiness, and numbness), “sickness symptom cluster” (pain, dry mouth, and disturbed sleep), and “gastrointestinal symptom cluster” (vomiting and nausea), respectively. The variance contribution of each symptom cluster was 22.4, 19.8, 16.2, and 12.3%, respectively (Table 3).

**Table 3:** Exploratory factor analysis of symptoms (*N* = 286).

**Cluster**	**Factor loading**			
	**Factor 1**	**Factor 2**	**Factor 3**	**Factor 4**
**Psychological status**				
Distress	0.836			
Sadness	0.823			
Lack of appetite	0.820			
Fatigue	0.754			
**Therapy side-effect**				
Forgetfulness		0.842		
Shortness of breath		0.799		
Drowsiness		0.771		
Numbness		0.697		
**Sickness**				
Pain			0.821	
Dry mouth			0.804	
Disturbed sleep			0.745	
**Gastrointestinal**				
Vomiting				0.897
Nausea				0.763
Explained variance (%)	22.4	19.8	16.2	12.3
Cumulative variance (%)	22.4	42.2	58.4	70.7

*Extraction method: Principal component analysis; Rotation method: Varimax with Kaiser normalization. Rotation converged after five iterations*.

### Correlation Analysis

Relationships among symptom clusters, illness perceptions, and QoL are presented in Table 4. The results of the correlation analysis revealed that symptom clusters (r = −0.40, *P* < 0.01) and illness perceptions (r = −0.57, *P* < 0.01) were negatively correlated with global QoL, while symptom clusters were positively correlated with illness perceptions (r = 0.30, *P* < 0.01). Consequently, we used SEM to evaluate the relationships among these variables and test the mediating effect of illness perceptions between symptom clusters and QoL.

**Table 4:** Correlations between symptom clusters, illness perception, and quality of life (*N* = 286).

**Variable**	**Mean**	**SD**	**1**	**2**	**3**	**4**	**5**	**6**	**7**	**8**	**9**	**10**	**11**	**12**	**13**	**14**	**15**
1 SC-PS	13.22	6.49	-														
2 SC-TSE	8.45	4.46	0.33^**^	-													
3 SC-S	9.19	4.12	0.40^**^	0.19^**^	-												
4 SC-G	6.33	3.63	0.49^**^	0.28^**^	0.44^**^	-											
5 SC-T	37.18	13.59	0.84^**^	0.62^**^	0.67^**^	0.73^**^	-										
6 IP-CIR	22.84	5.27	0.26^**^	0.15^*^	0.17^**^	0.21^**^	0.28^**^	-									
7 IP-ER	11.56	2.83	0.16^**^	0.06	0.14^*^	0.24^**^	0.20^**^	0.37^**^	-								
8 IP-IC	3.67	1.77	0.12^*^	−0.03	0.06	0.01	0.07	0.24^**^	−0.17^**^	-							
9 IP-T	38.07	7.26	0.28^**^	0.13^*^	0.19^**^	0.24^**^	0.30^**^	0.93^**^	0.62^**^	0.35^**^	-						
10 QoL-PWB	18.78	4.50	−0.47^**^	−0.14^*^	−0.22^**^	−0.26^**^	−0.41^**^	−0.52^**^	−0.17^**^	−0.21^**^	−0.49^**^	-					
11 QoL-SWB	14.43	3.53	−0.28^**^	−0.06	−0.12^*^	−0.19^**^	−0.24^**^	−0.36^**^	−0.31^**^	0.03	−0.38^**^	0.37^**^	−				
12 QoL-EWB	15.33	4.10	−0.41^**^	−0.17^**^	−0.19^**^	−0.27^**^	−0.38^**^	−0.51^**^	−0.28^**^	−0.11	−0.50^**^	0.66^**^	0.49^**^	−			
13 QoL-FWB	12.53	4.14	−0.45^**^	−0.13^*^	−0.23^**^	−0.23^**^	−0.39^**^	−0.44^**^	−0.15^**^	−0.23^**^	−0.43^**^	0.60^**^	0.43^**^	0.60^**^	-		
14 QoL-CCS	38.17	5.48	−0.19^**^	−0.10	−0.09	−0.10	−0.18^**^	−0.39^**^	−0.30^**^	−0.01	−0.40^**^	0.45^**^	0.47^**^	0.49^**^	0.41^**^	-	
15 QoL-T	99.24	16.83	−0.46^**^	−0.16^**^	−0.21^**^	−0.26^**^	−0.40^**^	−0.57^**^	−0.31^**^	−0.13^*^	−0.57^**^	0.80^**^	0.69^**^	0.83^**^	0.78^**^	0.77^**^	-

*SC, Symptom clusters; PS, Psychological status; TSE, Therapy side-effect; S, Sickness; G, Gastrointestinal; T, Total; IP, Illness perceptions; CIR, Cognitive illness representations; ER, Emotional representations; IC, Illness comprehensibility; QoL, Quality of life; PWB, Physical well-being; SWB, Social/family well-being; EWB, Emotional well-being; FWB, Functional well being; CCS, Cervical cancer subscales.*

* *p < 0.05,*

** *p < 0.01*.

### Mediation Effects

Regression analysis was carried out to assess the influence of sociodemographic and clinical variables on QoL before constructing SEM. The results showed that only monthly income per capita (β = 0.146, *p* = 0.014) significantly affect QoL, which were incorporated into the estimated model as control variables.

**Figure 1:**
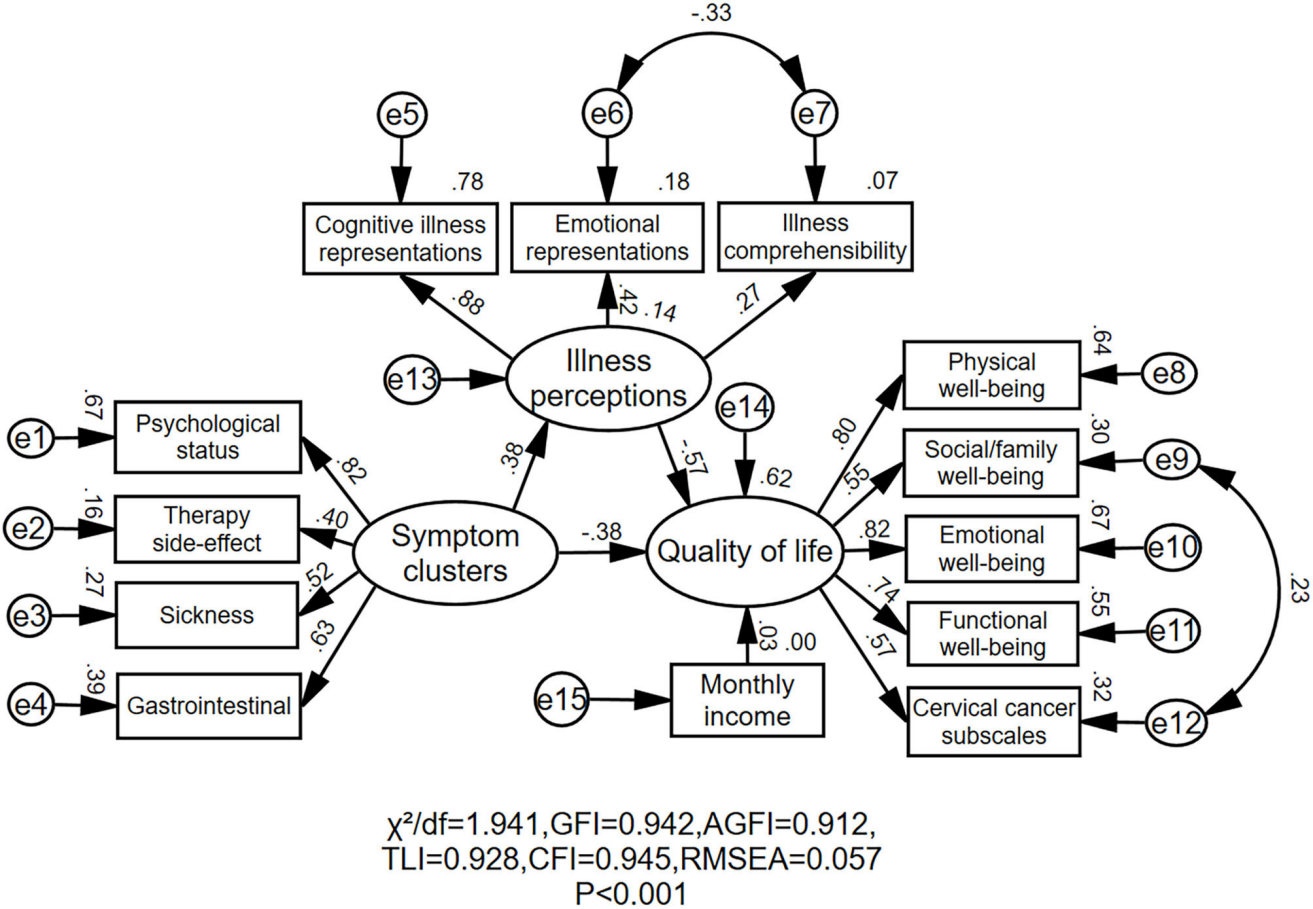
Final structural model. The Standardized path coefficients are presented for each pathway. χ^2^: Chi-square; df, degrees of freedom; GFI, goodness-of-fit index; AGFI, adjusted goodness-of-fit index; TLI, Tucker–Lewis Index; CFI, comparative fit index; RMSEA, root mean square error of approximation; e, error.

Figure 1 shows the final structural equation model and the standardized path loadings, all of which are significant with *P* < 0.001. For several modification indices greater than 10, further modifications were suggested to improve the model fit of the original model. The final best-fitting model was described below: χ^2^/df = 1.941, *P* < 0.001; GFI = 0.942, AGFI = 0.912, TLI = 0.928, CFI = 0.945, and RMSEA = 0.057. Symptom clusters and illness perceptions explained about 63% of the variation in the QoL.

As shown in Table 5, symptom clusters had significant direct effects on illness perceptions (β = 0.38, *P* < 0.001) and QoL (β = −0.38, *P* < 0.001). Illness perceptions had a significant direct effect on QoL (β = −0.57, *P* < 0.001). The indirect effect of symptom clusters on QoL was significant when illness perceptions were included in the final model as a mediator variable (β = −0.21, *P* < 0.001). The total effect of symptom clusters on QoL was (β = −0.60, *P* < 0.001). Therefore, illness perceptions had a partial mediating variable role in the effect of symptom clusters on QoL: the mediating effect amounted to 36.1%.

**Table 5:** The effects of explanatory variables on dependent variables (*N* = 286).

**Structural path**	**β (95% CI)**	**SE**	***P*-value**
**Direct effects**			
Symptom clusters → illness perceptions	0.38 (0.212, 0.536)	0.083	<0.001
Symptom clusters → QoL	−0.38 (−0.512, −0.218)	0.074	<0.001
Illness perceptions → QoL	−0.57 (−0.699, −0.430)	0.069	<0.001
**Indirect effects**	−0.21 (−0.341, −0.118)	0.056	<0.001
**Total effects**	−0.59 (−0.699, −0.454)	0.063	<0.001

*CI, Confidence interval; SE, Standard error; QoL, Quality of life*.

## Discussion

To the best of our knowledge, this is the first study to explore the relationship between symptom clusters and QoL through the mediating effect of illness perceptions in cervical cancer patients receiving CCRT according to the CSM. Our main findings were: (1) four symptom clusters were identified in this population; (2) symptom clusters and illness perceptions were significantly negatively associated with QoL; (3) symptom clusters were not only directly associated with QoL but also indirectly associated with QoL through illness perceptions. The results revealed initial evidence for the mediation of illness perceptions in the effects of symptom clusters on QoL.

In the current study, we kept 13 symptoms to carry out EFA and extracted four symptom clusters. Our research further confirmed that fatigue was the most prevalent and serious symptom in cancer patients, which was similar to the results of previous studies (40, 41). Cancer-related fatigue, with a prevalence rate of 14.0 to 100.0%, is one of the most common side effects of cancer survivors, which could significantly interfere with physical, emotional, and cognitive functions (42, 43). These findings emphasize the importance of evaluating and managing fatigue in cervical cancer patients during CCRT. The four symptom clusters were psychological status symptom cluster, therapy side-effect symptom cluster, sickness symptom cluster, and gastrointestinal symptom cluster. Our study supported that the gastrointestinal symptom cluster was one of the most common symptom clusters, which had been confirmed by several studies (18, 44, 45). A possible explanation for the mechanism of the gastrointestinal symptom cluster is that chemotherapeutic agents may activate gag reflex by binding to 5-HT3 receptors on vagus nerve endings to transmit impulses to the chemoreceptor trigger zone located in the medulla oblongata (46). Significantly, in previous studies, the number of symptom clusters and the symptoms in each group were not exactly the same. For example, Wang et al. investigated four symptom clusters (gastrointestinal, mood-cognitive, sickness-behavior, and pain-related symptom cluster) in cervical cancer patients using the MDASI (19). Pozzar et al. identified three symptom clusters (hormonal, respiratory, and weight change symptom cluster) across all the dimensions of occurrence, severity, and distress in gynecologic cancer patients undergoing chemotherapy using the Memorial Symptom Assessment Scale (MSAS) (40). In addition to the latent impact of the research population and instruments, the main reason for the inconsistencies is that symptoms could change with the progress of treatment undergoing CCRT (47). Thus, health care workers should put particular emphasis on the identification of symptom clusters, especially problems with fatigue, to guide prompt and effective symptom management interventions.

Our study demonstrated that symptom clusters were negatively associated with QoL, that was, greater symptom burden led to a poor QoL. A recent study from Africa reported that symptom experience significantly negatively affected QoL among cervical cancer patients (48). In addition, similar results have also been determined in other cancer patients. Authors of a previous study have shown that in breast cancer patients, the higher group within the physical–gastrointestinal symptom cluster had a poor QoL in all domains than the lower group, while in the psychological–general symptom cluster, the higher group had a poor QoL in all domains except sexual functioning, which partially supported our findings that greater symptom distress resulted in a worse QoL (18). Nevertheless, Li et al.'s research on head and neck cancer patients with endotracheal tubes revealed that there was no significant difference in the scores of QoL between the high and low severity groups of the digestive symptom cluster (47). These contradictory results may be related to disease characteristics, treatment modalities, and the different study periods investigated (49, 50). As there have been few studies of symptom clusters focused on cervical cancer, our study provides new evidence to support that symptom clusters might be reliable predictors of QoL in this population.

The results also revealed that illness perceptions had a significant negative effect on QoL in cervical cancer patients undergoing CCRT. Agreeing with previous studies' findings, the current study showed the negative role that illness perceptions could play in QoL (24, 51, 52). According to Leventhal's theoretical framework, the CSM emphasizes that illness perceptions, as emotional representations, together with cognitive representations, constitute the core for individuals to predict adherence to treatments, lifestyle changes, and choices of coping style for managing health threats (53). A higher perception of disease, which is a worse deviation from the normal self, is associated with poor QoL and higher mortality (52, 53). Specially, the intimate nature and perceived stigma of gynecologic cancers may reduce patients' willingness to seek help from a doctor, which would further affect global QoL and well-being (54, 55). One study reported that the psychological distress caused by illness perceptions could last for 5 to 10 years among gynecologic cancers survivors (56). In other words, illness perceptions were statistically significant predictors of survivors' long-term well-being. However, de Rooij et al. confirmed that threatening illness perceptions had negative impacts on physical, social, and psychological well-being, but it was yet unclear whether these effects would persist in the long term (23). In line with these cross-sectional findings, our study suggested that the global QoL had deteriorate with the increase of illness perceptions. Hence, medical staff should focus on reducing illness perceptions and assisting patients in developing individual coping skills to improve mental health and QoL in cervical cancer patients receiving CCRT.

This current study is the first to confirm the mediating effect of illness perceptions on the relationship between symptom clusters and QoL. In other words, illness perceptions strengthened the negative effect of symptom clusters on QoL. As previously addressed in several chronic diseases, the literature had proved that illness perceptions played an important mediating role between the symptom distress and patients' physical and social well-being (27–29, 57). According to the CSM theory, patients would generate cognitive representations based on information regarding their physical characteristics of the disease (e.g., severity of symptoms) and previous experience (e.g., treatment modalities) when they perceived a potential threat to health (20). Then, these beliefs could interfere with coping strategies and illness outcomes such as psychological health and QoL. Individuals who experience severe symptom distress could develop more pessimistic illness perceptions of their illness, which might translate into negative psychological well-being and ultimately lead to a deterioration in the QoL (57). Notably, Pereira et al. determined that the association between breast symptoms and QoL was totally mediated by illness perceptions, while our study proved that illness perceptions only played a partial role in mediating the impact between symptom distress and QoL (27). This suggests a specific impact of symptom severity on QoL, which is consistent with the results by Rha et al. (58). In addition, the effects of sociodemographic and clinical variables on QoL were controlled in our final model. The results of this study revealed that monthly income appeared to positively correlated with QoL, which echoes the previous study (4, 59). The possible explanation is that the choice of treatment modalities was affected by the economic status of the patients, resulting in differences in health outcomes and QoL.

### Study Strengths and Limitations

This was a multicenter study with an adequate sample size and SEM was first used to examine the mediating role of illness perceptions in the relationships between symptom clusters and QoL among cervical cancer patients undergoing CCRT. The following limitations need to be considered: (a) the majority of data were derived from participant self-report; (b) samples were collected from three hospitals using convenient sampling. Thus, the results will not necessarily represent all cervical cancer patients undergoing CCRT in China; (c) there may be a natural bias as the participants with different chemoradiotherapy regimens, treatment courses, and stages were included. (d) the findings of this study could only be interpreted cautiously as associations, as we cannot determine causality through a cross-sectional design. Further randomized, prospective, and longitudinal researches are needed to identify more stable symptom clusters and explore the long-term impacts of symptom clusters and illness perceptions on QoL.

## Conclusion

The results further confirmed that symptom clusters have a direct impact on QoL and illness perceptions play a mediating role in the relationship between symptom clusters and QoL, which have never been deeply explored in cervical cancer patients. Illness perceptions reinforce the negative effects of symptom clusters on QoL. This would prompt healthcare providers to focus not only on symptom distress but also on illness perceptions. Hence, interventions aimed at ameliorating symptom clusters and decreasing illness perceptions could be necessary to improve QoL among cervical cancer patients receiving CCRT.

## Data Availability Statement

The raw data supporting the conclusions of this article will be made available by the authors, without undue reservation.

## Ethics Statement

The studies involving human participants were reviewed and approved by the Local Ethics Committee of Shandong University. The patients/participants provided their written informed consent to participate in this study.

## Author Contributions

LZ and JW conceived and designed this study. TC, MT, QZ, and JR contributed to the data collection and interpretation. LZ wrote the main manuscript text. LZ, JW, and JR edited and revised the manuscript. All authors analyzed the data and approved final manuscript.

## Funding

This research was supported by Sichuan Science and Technology Program No. 2020YFS0049.

## Conflict of Interest

The authors declare that the research was conducted in the absence of any commercial or financial relationships that could be construed as a potential conflict of interest.

## Publisher's Note

All claims expressed in this article are solely those of the authors and do not necessarily represent those of their affiliated organizations, or those of the publisher, the editors and the reviewers. Any product that may be evaluated in this article, or claim that may be made by its manufacturer, is not guaranteed or endorsed by the publisher.

## References

[1] Bray F Ferlay J Soerjomataram I Siegel RL Torre LA Jemal A. Global cancer statistics 2018: GLOBOCAN estimates of incidence and mortality worldwide for 36 cancers in 185 countries. CA Cancer J Clin. (2018) 68:394–424. 10.3322/caac.21492 30207593

[2] Arbyn M Weiderpass E Bruni L de Sanjose S Saraiya M Ferlay J. Estimates of incidence and mortality of cervical cancer in 2018: a worldwide analysis. Lancet Global Health. (2020) 8:E191–203. 10.1016/S2214-109X(19)30482-6 31812369 PMC7025157

[3] Potter R Tanderup K Kirisits C de Leeuw A Kirchheiner K Nout R. The EMBRACE II study: the outcome and prospect of two decades of evolution within the GEC-ESTRO GYN working group and the EMBRACE studies. Clin Transl Radiat Oncol. (2018) 9:48–60. 10.1016/j.ctro.2018.01.001 29594251 PMC5862686

[4] Thapa N Maharjan M Xiong Y Jiang D Thi-Phuong N Petrini MA. Impact of cervical cancer on quality of life of women in Hubei, China. Sci Rep. (2018) 8:11993. 10.1038/s41598-018-30506-6 30097622 PMC6086893

[5] Koh W-J Abu-Rustum NR Bean S Bradley K Campos SM Cho KR. Cervical cancer, version 3.2019. J Natl Compr Canc Netw. (2019) 17:64–84. 10.6004/jnccn.2019.0001 30659131

[6] Greimel ER Winter R Kapp KS Haas J. Quality of life and sexual functioning after cervical cancer treatment: a long-term follow-up study. Psychooncology. (2009) 18:476–82. 10.1002/pon.1426 18702067

[7] Bjelic-Radisic V Jensen PT Vlasic KK Waldenstrom A-C Singer S Chie W. Quality of life characteristics inpatients with cervical cancer. Eur J Cancer. (2012) 48:3009–18. 10.1016/j.ejca.2012.05.011 22683166

[8] Li C-C Chang T-C Tsai Y-F Chen L. Quality of life among survivors of early-stage cervical cancer in Taiwan: an exploration of treatment modality differences. Qual Life Res. (2017) 26:2773–82. 10.1007/s11136-017-1619-0 28608151

[9] Kim J-EE Dodd MJ Aouizerat BE Jahan T Miaskowski C. A review of the prevalence and impact of multiple symptoms in oncology patients. J Pain Symptom Manage. (2009) 37:715–36. 10.1016/j.jpainsymman.2008.04.018 19019626 PMC2688644

[10] Tagami K Kawaguchi T Miura T Yamaguchi T Matsumoto Y Watanabe YS. The association between health-related quality of life and achievement of personalized symptom goal. Support Care Cancer. (2020) 28:4737–43. 10.1007/s00520-020-05316-0 31970517

[11] del Carmen MG Rice LW. Management of menopausal symptoms in women with gynecologic cancers. Gynecol Oncol. (2017) 146:427–35. 10.1016/j.ygyno.2017.06.013 28625396

[12] Frumovitz M Obermair A Coleman RL Pareja R Lopez A Ribero R. Quality of life in patients with cervical cancer after open versus minimally invasive radical hysterectomy (LACC): a secondary outcome of a multicentre, randomised, open-label, phase 3, non-inferiority trial. Lancet Oncol. (2020) 21:851–60. 10.1016/S1470-2045(20)30081-4 32502445 PMC9762514

[13] Fleming ND Ramirez PT Soliman PT Schmeler KM Chisholm GB Nick AM. Quality of life after radical trachelectomy for early-stage cervical cancer: a 5-year prospective evaluation. Gynecol Oncol. (2016) 143:596–603. 10.1016/j.ygyno.2016.10.012 27742473 PMC5439265

[14] Miaskowski C Aouizerat BE Dodd M Cooper B. Conceptual issues in symptom clusters research and their implications for quality-of-life assessment in patients with cancer. J Natl Cancer Inst Monogr. (2007) 37:39–46. 10.1093/jncimonographs/lgm003 17951230

[15] Yu DS Li PW Chong SO. Symptom cluster among patients with advanced heart failure: a review of its manifestations and impacts on health outcomes. Curr Opin Support Palliat Care. (2018) 12:16–24. 10.1097/SPC.0000000000000316 29176333

[16] Nho J-H Kim SR Nam J-H. Symptom clustering and quality of life in patients with ovarian cancer undergoing chemotherapy. Eur J Oncol Nurs. (2017) 30:8–14. 10.1016/j.ejon.2017.07.007 29031318

[17] Choi S Ryu E. Effects of symptom clusters and depression on the quality of life in patients with advanced lung cancer. Eur J Cancer Care. (2018) 27:e12508. 10.1111/ecc.12508 27112232

[18] Nho J-H Kim S-R Park M-H Kweon S-S. Symptom clusters and quality of life in breast cancer survivors after cancer treatment in a tertiary hospital in Korea. Eur J Cancer Care. (2018) 27:e12919. 10.1111/ecc.12919 30253019

[19] Wang CL Wu WY Lou HM Wang HM Liang GM Xia LY. Analysis of symptom clusters in Chinese cervical cancer patients undergoing radiotherapy, chemoradiotherapy, or postoperative chemoradiotherapy. Eur J Gynaecol Oncol. (2017) 38:398–403. 10.12892/ejgo3618.2017 29693881

[20] Leventhal H Safer MA Panagis DM. The impact of communications on the self-regulation of health beliefs, decisions, and behavior. Health Educ Q. (1983) 10:3–29. 10.1177/109019818301000101 6629788

[21] Hagger MS Orbell S. A meta-analytic review of the common-sense model of illness representations. Psychol Health. (2003) 18:141–84. 10.1080/08870440310008132128805401

[22] Hopman P Rijken M. Illness perceptions of cancer patients: relationships with illness characteristics and coping. Psychooncology. (2015) 24:11–18. 10.1002/pon.3591 24891136

[23] de Rooij BH Ezendam NPM Nicolaije KAH Lodder P Vos MC Pijnenborg JMA. Survivorship care plans have a negative impact on long-term quality of life and anxiety through more threatening illness perceptions in gynecological cancer patients: the ROGY care trial. Qual Life Res. (2018) 27:1533–44. 10.1007/s11136-018-1825-4 29511906 PMC5951872

[24] Sayilan AA Dogan MD. Illness perception, perceived social support and quality of life in patients with diagnosis of cancer. Eur J Cancer Care. (2020) 29:e13252. 10.1111/ecc.13252 32495471

[25] Zhang Z Yang L Xie D Wang Y Bi L Zhang T. Illness perceptions are a potential predictor of psychological distress in patients with non-muscle-invasive bladder cancer: a 12-month prospective, longitudinal, observational study. Psychol Health Med. (2020) 25:969–79. 10.1080/13548506.2019.1707242 31868002

[26] Xiong NN Wei J Ke MY Hong X Li T Zhu LM. Illness perception of patients with functional gastrointestinal disorders. Front Psychiatry. (2018) 9:122. 10.3389/fpsyt.2018.00122 29706904 PMC5906533

[27] Pereira M Moreira CS Nogueira-Silva C Izdebski P Grata Pereira M. Breast cancer post-surgical impact on women ' s quality of life during chemotherapy treatment: a structural equation modelling approach. Eur J Cancer Care. (2021) 30:e13349. 10.1111/ecc.13349 33159394

[28] Woodhouse S Hebbard G Knowles SR. Exploring symptom severity, illness perceptions, coping styles, and well-being in gastroparesis patients using the common sense model. Dig Dis Sci. (2018) 63:958–65. 10.1007/s10620-018-4975-x 29468373

[29] De Gucht V. Illness perceptions mediate the relationship between bowel symptom severity and health-related quality of life in IBS patients. Qual Life Res. (2015) 24:1845–56. 10.1007/s11136-015-0932-8 25663636 PMC4493794

[30] de Castro EK Peuker AC Lawrenz P Figueiras MJ. Illness perception, knowledge and self-care about cervical cancer. Psicol Reflexao E Crit. (2015) 28:483–9. 10.1590/1678-7153.20

[31] Lei Pa Wu Q. Introduction to structural equation modeling: issues and practical considerations. Educ Meas Issues Pract. (2007) 26:33–43. 10.1111/j.1745-3992.2007.00099.x

[32] Eraj SA Jomaa MK Rock CD Mohamed ASR Smith BD Smith JB. Long-term patient reported outcomes following radiation therapy for oropharyngeal cancer: cross-sectional assessment of a prospective symptom survey in patients ≥65 years old. Radiat Oncol. (2017) 12:150. 10.1186/s13014-017-0878-9 28888224 PMC5591495

[33] Wang XS Wang Y Guo H Mendoza TR Hao XS Cleeland CS. Chinese version of the M.D. Anderson Symptom Inventory - Validation and application of symptom measurement in cancer patients. Cancer. (2004) 101:1890–901. 10.1002/cncr.20448 15386315

[34] Broadbent E Petrie KJ Main J Weinman J. The brief illness perception questionnaire. J Psychosom Res. (2006) 60:631–7. 10.1016/j.jpsychores.2005.10.020 16731240

[35] Zhang N Fielding R Soong I Chan KK Lee C Ng A. Psychometric assessment of the Chinese version of the brief illness perception questionnaire in breast cancer survivors. PLoS ONE. (2017) 12:e0174093. 10.1371/journal.pone.0174093 28319160 PMC5358881

[36] Cella DF Tulsky DS Gray G Sarafian B Linn E Bonomi A. The functional assessment of cancer-therapy scale - development and validation of the general measure. J Clin Oncol. (1993) 11:570–9. 10.1200/JCO.1993.11.3.570 8445433

[37] Ding Y Hu Y Hallberg IR. Psychometric properties of the Chinese version of the Functional Assessment of Cancer Therapy-Cervix (FACT-Cx) measuring health-related quality of life. Health Qual Life Outcomes. (2012) 10:124. 10.1186/1477-7525-10-124 23031680 PMC3503601

[38] Joliffe IT Morgan BJ. Principal component analysis and exploratory factor analysis. Stat Methods Med Res. (1992) 1:69–95. 10.1177/096228029200100105 1341653

[39] McDonald RP Ho MH. Principles and practice in reporting structural equation analyses. Psychol Methods. (2002) 7:64–82. 10.1037/1082-989X.7.1.64 11928891

[40] Pozzar RA Hammer MJ Cooper BA Kober KM Chen L-M Paul SM. Symptom clusters in patients with gynecologic cancer receiving chemotherapy. Oncol Nurs Forum. (2021) 48:441–52. 10.1188/21.ONF.441-452 34143001

[41] Ma Y He B Jiang M Yang Y Wang C Huang C. Prevalence and risk factors of cancer-related fatigue: a systematic review and meta-analysis. Int J Nurs Stud. (2020) 111:103707. 10.1016/j.ijnurstu.2020.103707 32920423

[42] Hanna EY Mendoza TR Rosenthal DI Gunn GB Sehra P Yucel E. The symptom burden of treatment-naive patients with head and neck cancer. Cancer. (2015) 121:766–73. 10.1002/cncr.29097 25369213 PMC4339345

[43] Wang S-H He G-P Jiang P-L Tang L-L Feng X-M Zeng C. Relationship between cancer-related fatigue and personality in patients with breast cancer after chemotherapy. Psychooncology. (2013) 22:2386–90. 10.1002/pon.3303 23674435

[44] Li N Wu J Zhou J Wu C Dong L Fan W. Symptom clusters change over time in patients with lung cancer during perichemotherapy. Cancer Nurs. (2021) 44:272–80. 10.1097/NCC.0000000000000787 32022780

[45] Pozzar RA Hammer MJ Cooper BA Kober KM Chen L-M Paul SM. Stability of symptom clusters in patients with gynecologic cancer receiving chemotherapy. Cancer Nurs. (2021). 10.1097/NCC.0000000000000988 [Epub ahead of print]. 34560709 PMC8940749

[46] Cherwin CH. Gastrointestinal symptom representation in cancer symptom clusters: a synthesis of the literature. Oncol Nurs Forum. (2012) 39:157–65. 10.1188/12.ONF.157-165 22374489 PMC3365541

[47] Li Y Li X Mao C Lv G Xie Z Jiang H. Symptom clusters in head and neck cancer patients with endotracheal tube: which symptom clusters are independently associated with health-related quality of life? Eur J Oncol Nurs. (2020) 48:101819. 10.1016/j.ejon.2020.101819 32937263

[48] Araya LT Fenta TG Sander B Gebremariam GT Gebretekle GB. Health-related quality of life and associated factors among cervical cancer patients at Tikur Anbessa specialized hospital, Addis Ababa, Ethiopia. Health Qual Life Outcomes. (2020) 18:72. 10.1186/s12955-020-01319-x 32178681 PMC7076924

[49] Ren H Tang P Zhao Q Ren G. Symptom clusters and related factors in bladder cancer patients three months after radical cystectomy. BMC Urol. (2017) 17:65. 10.1186/s12894-017-0255-x 28835243 PMC5569499

[50] Klapheke AK Keegan THM Ruskin R Cress RD. Changes in health-related quality of life in older women after diagnosis with gynecologic cancer. Gynecol Oncol. (2020) 156:475–481. 10.1016/j.ygyno.2019.11.033 31806400

[51] Fanakidou I Zyga S Alikari V Tsironi M Stathoulis J Theofilou P. Mental health, loneliness, and illness perception outcomes in quality of life among young breast cancer patients after mastectomy: the role of breast reconstruction. Qual Life Res. (2018) 27:539–43. 10.1007/s11136-017-1735-x 29119452

[52] de Rooij BH Thong MSY van Roij J Bonhof CS Husson O Ezendam NPM. Optimistic, realistic, and pessimistic illness perceptions; quality of life; and survival among 2457 cancer survivors: the population-based PROFILES registry. Cancer. (2018) 124:3609–17. 10.1002/cncr.31634 30192384

[53] Leventhal H Phillips LA Burns E. The Common-Sense Model of Self-Regulation (CSM): a dynamic framework for understanding illness self-management. J Behav Med. (2016) 39:935–46. 10.1007/s10865-016-9782-2 27515801

[54] Crist JV Grunfeld EA. Factors reported to influence fear of recurrence in cancer patients: a systematic review. Psychooncology. (2013) 22:978–86. 10.1002/pon.3114 22674873

[55] Handschel J Naujoks C Kuebler NR Krueskemper G. Fear of recurrence significantly influences quality of life in oral cancer patients. Oral Oncol. (2012) 48:1276–80. 10.1016/j.oraloncology.2012.06.015 22818822

[56] Tsai L-Y Lee S-C Wang K-L Tsay S-L Tsai J-M. A correlation study of fear of cancer recurrence, illness representation, self-regulation, and quality of life among gynecologic cancer survivors in Taiwan. Taiwan J Obstet Gynecol. (2018) 57:846–52. 10.1016/j.tjog.2018.10.014 30545539

[57] Zhang M Hong L Zhang T Lin Y Zheng S Zhou X. Illness perceptions and stress: mediators between disease severity and psychological well-being and quality of life among patients with Crohn's disease. Pat Prefer Adher. (2016) 10:2387–96. 10.2147/PPA.S118413 27920505 PMC5125764

[58] Rha SY Lee J. Symptom clusters during palliative chemotherapy and their influence on functioning and quality of life. Support Care Cancer. (2017) 25:1519–27. 10.1007/s00520-016-3545-z 28032218

[59] Fu L Feng X Jin Y Lu Z Li R Xu W. Symptom clusters and quality of life in gastric cancer patients receiving chemotherapy. J Pain Symptom Manage. (2021). 10.1016/j.jpainsymman.2021.09.003 [Epub ahead of print]. 34537311

